# Single-cell sequencing techniques from individual to multiomics analyses

**DOI:** 10.1038/s12276-020-00499-2

**Published:** 2020-09-15

**Authors:** Yukie Kashima, Yoshitaka Sakamoto, Keiya Kaneko, Masahide Seki, Yutaka Suzuki, Ayako Suzuki

**Affiliations:** 1 Department of Computational Biology and Medical Sciences, Graduate School of Frontier Sciences, The University of Tokyo, Chiba, Japan; 2 Division of Translational Genomics, Exploratory Oncology Research & Clinical Trial Center, National Cancer Center, Chiba, Japan

**Keywords:** RNA sequencing, Next-generation sequencing

## Abstract

Here, we review single-cell sequencing techniques for individual and multiomics profiling in single cells. We mainly describe single-cell genomic, epigenomic, and transcriptomic methods, and examples of their applications. For the integration of multilayered data sets, such as the transcriptome data derived from single-cell RNA sequencing and chromatin accessibility data derived from single-cell ATAC-seq, there are several computational integration methods. We also describe single-cell experimental methods for the simultaneous measurement of two or more omics layers. We can achieve a detailed understanding of the basic molecular profiles and those associated with disease in each cell by utilizing a large number of single-cell sequencing techniques and the accumulated data sets.

## Single-cell sequencing: Greater insight through integrated data

Combining data from different single-cell sequencing techniques could greatly improve understanding of the molecular profiles associated with disease. Sequencing studies provide valuable insights into diseased and healthy states at a single-cell level, for example the evolutionary paths of brain tumors and cancerous mutations. Ayako Suzuki at the University of Tokyo in Chiba, Japan, and co-workers examined the challenges of integrating data from various experimental and computational single-cell sequencing methods. These methods usually determine the genomic, epigenomic (DNA modifications) or transcriptomic (messenger RNAs) state of a cell, and can be combined to create a detailed picture. Other ‘multiomics’ techniques provide multilayered information from the same cell. The researchers recommend detailed analysis of individual data layers prior to integration, and highlight emerging techniques that analyze larger tissue sections, thus retaining the temporal and spatial information around a cell.

## Main

### Introduction: single-cell sequencing analysis

Recently, single-cell sequencing technologies have been rapidly developed for observing the multilayered status of single cells. Single-cell sequencing has the power to elucidate genomic, epigenomic, and transcriptomic heterogeneity in cellular populations, and the changes at these levels. A large number of reports on this topic have been published worldwide from various regions. Under an international approach, the Human Cell Atlas (HCA; https://www.humancellatlas.org/) has been generating comprehensive molecular maps of all human cells^1^. The HCA platform utilizes single-cell sequencing techniques to obtain single-cell genomic information from healthy and diseased cells. To study all types of cells and omics layers, we should consider single-cell sequencing methods from both laboratory and clinical perspectives. In this review, we introduce basic information and describe several applications of single-cell sequencing techniques.

### Single-cell transcriptome sequencing

**Fig. 1:**
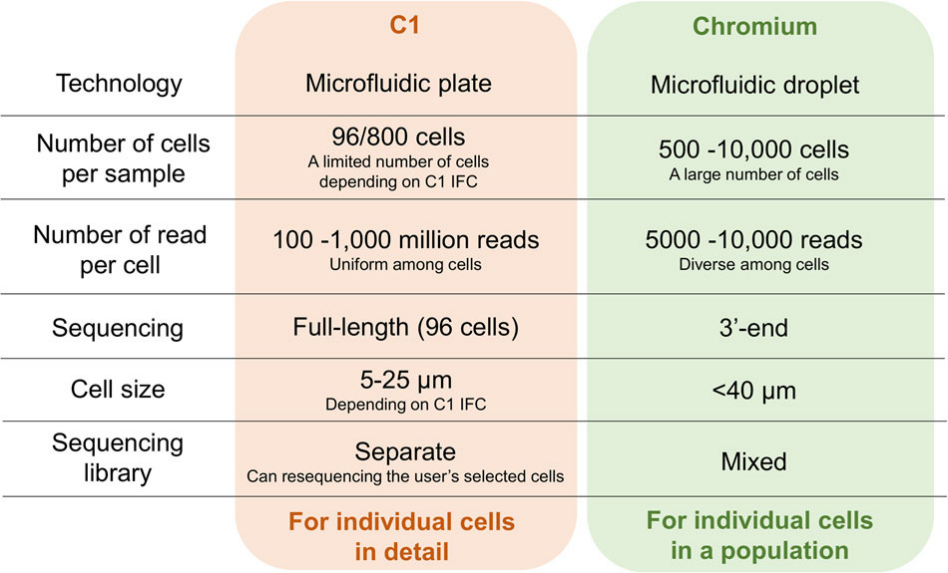
Comparison of scRNA-seq platforms. Characteristics of two major scRNA-seq platforms, C1 and Chromium.

Single-cell RNA sequencing (scRNA-seq)^2^ has been widely utilized worldwide. RNA-seq analysis conventionally measures transcripts in a mixture of cells (called a “bulk”). Bulk RNA-seq analysis allows the measurement of only the average transcript expression in a cell population. For example, in the RNA-seq of cancer tissue, transcripts from various types of cells, including tumor cells, immune cells, fibroblasts, and endothelial cells, are analyzed. To precisely understand the transcriptomic status of such heterogeneous cell populations, we can use scRNA-seq techniques (Table 1). For the analysis of tissues, cell dissociation is the most important step, as the conditions of this step directly affect the molecular profiles of cells, and the impacts of stress and damage depend on the cell type. For the measurement of transcripts in individual cells, reverse transcription (RT) and cDNA amplification must be performed from very small amounts of RNA. Smart-seq^3^ is a whole-transcriptome amplification (WTA) method that has been developed for full-length cDNA amplification with oligo-dT priming and template switching. Smart-seq2^4^, Quartz-Seq^5^, and CEL-seq^6^ have also been developed to stably measure mRNAs from a single cell. RamDa-seq^7^ detects non-poly(A) transcripts, including long noncoding RNAs and enhancer RNAs, in a single cell. Although diverse WTA methods exist, it is still difficult to perform scRNA-seq because the processing of hundreds to thousands of single cells and small amounts of liquid are conditions inherent to WTA methods. A number of methods for the simple procedure of scRNA-seq library construction have been reported. Several protocols based on microdroplet technology have been reported, such as Drop-Seq^8^ and DroNc-seq^9^. In these methods, a cell/nucleus, reaction liquid, and a barcoded bead are included in an oil droplet, and RT is conducted with molecular/cell barcoding within each oil droplet. On the other hand, in the microwell-seq^10^ approach, a cell and barcoded bead are isolated in a well. Nx1-seq^11^ and Seq-Well^12^ are reported to be portable, low-cost microwell-based platforms. These microdroplet- and microwell-based protocols enable the easy handling of thousands of single cells. For higher-throughput and lower-cost scRNA-seq analysis, sci-RNA-seq^13^ is a combinatorial indexing method (the recent version is sci-RNA-seq3^14^) that has been developed. Vendors have also developed automatic scRNA-seq platforms that can automatically conduct cell isolation, cell lysis, RT, and PCR amplification for each individual cell. The C1 Single-Cell Auto Prep system (Fluidigm) was launched in 2013. This platform enables the automatic isolation of 96 cells, cDNA synthesis, and amplification based on Smart-seq through microfluidics. C1-CAGE can also be conducted using the C1 system, which enables the profiling of the 5′ end of transcripts with strand information in a single cell^15^. Microdroplet-based systems such as Chromium (10× Genomics), ddSEQ (Bio-Rad/Illumina), Nadia (Dolomite), and inDrop (1CellBio) and microwell-based systems such as Rhapsody (BD) and ICELL8 (Takara) also exist. Researchers can select various methods and platforms for scRNA-seq (Table 1). However, problems such as limited cell capture, low RT efficiency, amplification bias and the requirement for a large number of sequencing reads remain, depending on the platform. Users should select appropriate methods of scRNA-seq for their sample type and research purpose (Fig. 1). For example, although only 96 cells can be analyzed per run with a C1 chip, as determined by its size, the C1/Smart-seq platform can be used to obtain full-length cDNA libraries for each cell separately and can perform additional sequencing of libraries in user-selected wells; therefore, we can obtain in-depth transcriptome information for each cell. Chromium enables the analysis of thousands of cells with simple protocols, while the libraries of selected cells cannot be reanalyzed because libraries are mixed after barcoding. In several studies, multiple methods have been applied to the same samples to complement the weak points of each method. We also conducted two types of scRNA-seq assays, bead-seq^16^ (involving a small number of cells and providing a large amount of information for each cell) and Chromium (involving many cells and providing less information per cell), to monitor transcriptome changes under anticancer drug treatment, and we extracted cells that exhibited an atypical expression pattern from bead-seq data and validated the cells in a larger population using Chromium data^17^. Furthermore, as scRNA-seq platforms and their consumables are frequently updated, we should be aware of their different versions. In particular, 10× Genomics updated the reagent kit from v2 to v3 for the Chromium system, and the detection sensitivity was greatly improved. They also recently modified the chip architecture of Next GEM technology. It is important to recognize the differences in the detection limits and dynamic ranges of gene expression levels when we compare or merge data across different versions of machines or kits.

**Table 1:** Single-cell transcriptome sequencing.

Method	Feature	References
Smart-seq	WTA method; template switching	^3^
CEL-seq	WTA method; in vitro transcription	^6^
Quartz-seq	WTA method; poly(A) tagging	^5^
C1-CAGE	5′-end RNA-seq	^15^
RamDa-seq	Total RNA-seq	^7^
Drop-seq	Microdroplet-based method	^8^
Microwell-seq	Microwell-based method	^10^

Recent scRNA-seq studies have been conducted in various research fields, such as immunology, developmental biology and oncology. In the field of cancer genomics, researchers have conducted the scRNA-seq of cancer cells and their surrounding stromal cells in the tumor microenvironment. Several groups have reported the scRNA-seq of brain tumors and revealed intratumor transcriptional heterogeneity and diverse evolutionary paths^18–20^. Tirosh et al.^21^ performed the first large-scale scRNA-seq study of the tumor ‘ecosystem’ and performed the scRNA-seq of CD45^+^ and CD45^−^ cells in 19 melanoma patients. They specifically elucidated different types of T cell exhaustion programs in each patient, which might be relevant for immunotherapy strategies. Chung et al.^22^ also focused on tumor and immune cells, including T cells, B cells and macrophages, in 11 breast cancer samples. Tumor-infiltrating lymphocytes in various types of cancers, such as hepatocellular carcinoma^23^, non-small-cell lung cancer^24^, and colon cancer, have also been targeted for scRNA-seq. In our group, to elucidate tumor evolution and the mechanism of acquired resistance to anticancer drugs, we conducted the scRNA-seq of lung cancer cell lines stimulated by receptor tyrosine kinase inhibitors. We observed different transcriptional responses to the drug among sensitive and insensitive cells^25^, and identified distinct transcriptional modules that might be associated with early resistance responses, such as dormancy^17^. The number of studies utilizing scRNA-seq is continuing to increase rapidly.

For the computational analysis of scRNA-seq data, we cannot simply use the bioinformatics approaches employed for bulk RNA-seq analysis because single-cell sequencing generates sparse multidimensional data. Seurat^26^ is an R package for scRNA-seq analysis that includes data filtering, normalization, scaling, dimensional reduction, clustering and visualization. To further analyze single-cell transcriptome data, various types of algorithms and tools have been developed, such as MAGIC^27^, SAVER^28^, and scImpute^29^ for imputation, Seurat CCA^30^ and ZINB-WaVE^31^ for batch effect removal, Monocle3^32^ and cellTree^33^ for pseudotime trajectory, and velocyte^34^ for RNA velocity and NicheNet^35^ for ligand–receptor interaction determination. There are an increasing number of tools for scRNA-seq analysis; as a result, we should select suitable tools for our own research purposes and data sets.

### Single-cell genome sequencing for understanding genetic heterogeneity

Single-cell genome sequencing enables the elucidation of genetic heterogeneity; thus, it can be used for the analysis of de novo germline mutations and somatic mutations in normal and cancer cells (Table 2). To uniformly amplify genomic DNA in individual cells, whole-genome amplification (WGA) methods have been developed^2^, such as multiple displacement amplification (MDA)^36^, multiple annealing and looping-based amplification cycles (MALBAC)^37^ and degenerate oligonucleotide-primed PCR (DOP-PCR)^38^. WGA is challenging for reasons such as the presence of only two copies of genomic DNA in human cells. This strategy occasionally misses an allele within a large genomic region (allelic dropout) and fails to achieve a uniform sequencing depth because of amplification bias. Care must be taken in the analysis of such genome sequencing data, especially in the detection of point mutations. Bioinformatics methods such as SCcaller^39^, Monovar^40^, LiRA^41^, and Conbase^42^ have been developed to detect single-nucleotide variants (SNVs) considering allelic dropout and amplification artifacts. For automatic library construction, the C1 system supports single-cell whole-genome and whole-exome sequencing. Furthermore, 10× Genomics recently released a copy number variant (CNV) solution for the Chromium system to profile copy numbers in single cells. The procedure for library construction is simplified, but a large number of sequencing reads are required, and the sequencing cost is very high.

**Table 2:** Single-cell genome sequencing.

Method	Feature	References
MDA	WGA method; isothermal amplification	^36^
DOP-PCR	WGA method; PCR-based	^38^
MALBAC	WGA method; hybrid	^37^

Single-cell genome sequencing reveals genetic heterogeneity. Mutations independently accumulate in cells and cause aging and diseases such as developmental diseases and cancers. Zhang et al.^43^ reported a single-cell whole-genome sequencing study of somatic mutations in B lymphocytes and observed the accumulation of somatic mutations with age and mutational signatures associated with the carcinogenesis of B cell cancers. They used the MDA method for WGA and obtained whole-genome sequencing data that covered approximately half of the genome regions at 20× and achieved greater sequencing depths. Neurogenerative diseases have also been analyzed through single-cell genome sequencing because most neurons exhibit longevity and cannot be renewed; thus, mutations tend to accumulate^44^. In a previous report^45^, a total of 159 single neurons from healthy and diseased individuals were sequenced to evaluate the accumulation of somatic mutations caused by aging or defects in DNA damage repair. Bae et al.^46^ also conducted the genome sequencing of single neurons from the prenatal brain and detected 200–400 SNVs per cell. In cancers, researchers have attempted to identify intratumor genetic heterogeneity generated during cancer evolution. Dr. Navin’s group reported a series of single-cell genome analyses of cancer cells, focusing on breast cancer cells in particular. They elucidated tumor progression through analyses of punctuated copy number evolution and the gradual evolution of point mutations by conducting single-cell genome sequencing and profiling mutations and CNVs in each individual cancer cell^47–49^. They also reported multiclonal invasion, which is a model of cancer evolution from ductal carcinoma in situ (DCIS), as an early stage in the progression of breast cancer to invasive ductal carcinoma (IDC)^50^. In another report, the adaptive selection of pre-existing clones was used as a model of chemoresistance to neoadjuvant therapy^51^. Furthermore, to understand the clonal evolution that leads to the acquisition of resistance to FLT3 inhibitors in acute myeloid leukemia (AML), McMahon et al.^52^ performed single-cell targeted DNA sequencing using the Tapestri platform (Mission Bio). They found that clones harboring RAS/MAPK mutations were selected after treatment with FLT3 inhibitors.

### Single-cell epigenome sequencing for detecting footprints of differentiation of individual cells

Single-cell sequencing technologies for studying epigenomics also exist (Table 3). By elucidating the epigenomic status of cells, such as DNA methylation and chromatin states, we can observe the cell lineage and differentiation state of individual cells. Single-cell DNA methylation profiling can be analyzed by single-cell bisulfite sequencing (scBS-seq)^53^ and single-cell reduced representation bisulfite sequencing (scRRBS)^54^.

**Table 3:** Single-cell epigenome sequencing.

Method	Target	Feature	References
scBS-seq	DNA methylation	Whole-genome BS-seq	^53^
scRRBS	DNA methylation	RRBS	^54^
scAba-seq	DNA methylation	5hmC sequencing	^77^
scATAC-seq	Chromatin accessibility	ATAC-seq	^58^
Drop-ChIP	Histone modification	ChIP-seq; microdroplet-based	^55^
scChIC-seq	Histone modification	Ab-Mnase	^78^
CUT&Tag	Histone modification	Ab + protein A-Tn5 transposase	^57^
Single-cell Hi-C	Chromatin structure	Hi-C	^79^

*Ab* antibody.

For the investigation of chromatin status, several methods can be used to measure the patterns of histone modifications in individual cells. Single-cell ChIP-seq can be conducted via a droplet microfluidics-based procedure known as Drop-ChIP^55^. This study reported the H3K4me2 and H3K4me3 patterns of mouse ES cells, embryonic fibroblasts and hematopoietic progenitors. Grosselin et al.^56^ recently conducted single-cell chromatin immunoprecipitation followed by sequencing (scChIP-seq) to analyze the H3K27me3 landscapes of patient-derived xenografts (PDXs) of breast cancers. They revealed differences between cells that were sensitive and resistant to chemotherapies and found that a fraction of sensitive tumors already harbored the distinct H3K27me3 patterns observed in resistant cells. Cleavage under targets and tagmentation (CUT&Tag)^57^ is another method used to profile chromatin components. First, an antibody identifies a target chromatin protein, such as a histone modification. Then, protein A and Tn5 transposase fusion proteins bind to the antibody and are tagged to the genomic regions where the target protein is bound.

Assay for transposase-accessible chromatin using sequencing (ATAC-seq) elucidates open chromatin patterns using a small number of cells. Open chromatin regions are tagged with sequencing adaptors by Tn5 transposase, amplified by PCR and sequenced. Several single-cell platforms, including the C1 and Chromium systems, enable single-cell ATAC-seq (scATAC-seq). In the C1 system, all steps of library preparation, from cell lysis to PCR amplification, are automatically conducted with microfluidics^58^. For the Chromium Single-Cell ATAC Solution approach, researchers must prepare isolated nuclei and conduct Tn5 tagmentation before separation in droplets. scATAC-seq is useful for analyzing transcriptional regulatory programs in mixed cell populations including various lineages and developmental stages, such as blood cells. Corces et al.^59^ reported the application of “enhancer cytometry” for the identification of cell types in a mixed population of blood cells using ATAC-seq data, which included the in silico deconvolution of cell types based on enhancer patterns. They constructed a regulatory map of hematopoiesis and elucidated the AML cell population with the projection of scATAC-seq data for validation.

### Proteomics analysis at the single-cell level

To comprehensively measure the expression patterns of each protein, researchers generally use mass spectrometry or flow cytometry rather than sequencing. Technical challenges related to factors such as the required sample amounts and detection coverage are encountered in the application of mass spectrometry to single-cell proteomics, such that various study groups are now making an effort to develop methods for measurement of more protein molecules using a lower sample input. In recent single-cell studies, CyToF, which is a method based on mass cytometry, has been used to analyze tens of surface and intracellular proteins by using antibodies tagged with metal labels. For immune cells, in particular, the profiling of cell surface proteins is useful for the classification of cell types. There have been many studies using CyToF, including general and cancer immunology studies, often in combination with scRNA-seq analysis.

### Integration of different layers of single-cell data sets

Single-cell sequencing enables the elucidation of the omics features of each layer of genomic, epigenomic and transcriptomic data. Many studies have attempted to integrate single-cell data sets that are independently obtained from multiple layers.

**Fig. 2:**
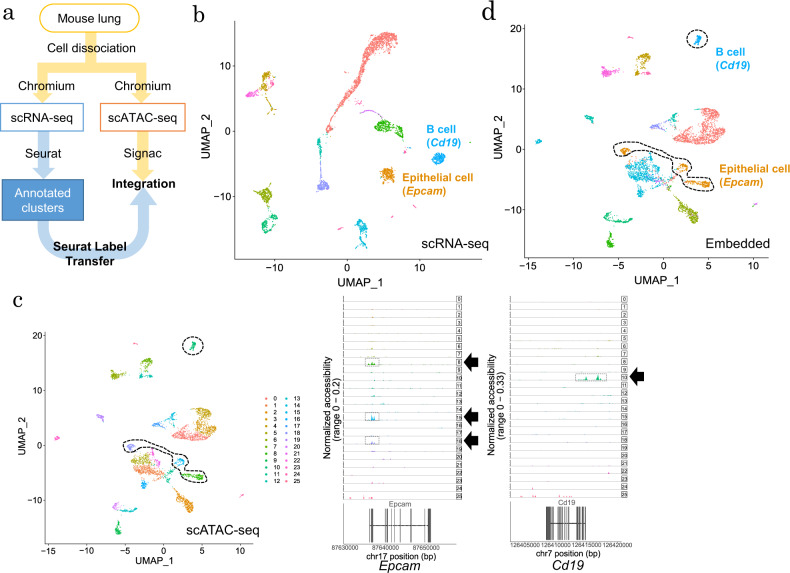
Integration of scRNA-seq and scATAC-seq in mouse lung cells. **a** The workflow for the integration of scRNA-seq and sATAC-seq. **b** 2D visualization of scRNA-seq clusters from mouse lungs. The UMAP figure was created with Seurat v3.1.2. The cell types in each cluster were identified on the basis of the expression levels of cell type-specific markers. The clusters with the same cell type annotation were merged. In this figure, clusters of epithelial cells with *Epcam* and B cells with *Cd19* were the focus. **c** 2D visualization of scATAC-seq clusters (left). The UMAP figure was created by using Signac v0.1.6. Coverage plots are shown for two marker genes (right). **d** UMAP visualization of scATAC-seq with Seurat Label Transfer from scRNA-seq data. The cell types in the scATAC-seq clusters were predicted by scRNA-seq annotation.

To integrate different layers of single-cell omics data, several computational methods have been developed, such as Seurat Label Transfer^60^ and LIGER^61^. To provide an overview multiomics single-cell analysis, we describe a representative case for analysis involving the mouse lung. As shown in Fig. 2, we conducted scRNA-seq and scATAC-seq of mouse lung cells using the Chromium system and tried to integrate the results using Seurat Label Transfer. We generated scRNA-seq data sets using Chromium after the dissociation of mouse lung tissue according to the manufacturer’s protocol. We also extracted nuclei from the mouse lung tissue for scATAC-seq. We used Cell Ranger and Cell Ranger ATAC, which are analytical pipelines provided by 10× Genomics, to extract matrices of RNA expression and open chromatin patterns from each data set for individual cells. For scRNA-seq, we used Seurat v3 and annotated cell subpopulations (clusters) according to known cell type markers, such as *Epcam* and *Cdh1* for epithelial cells and *Cd19* for B cells, following the filtering of low-quality data, dimensional reduction and clustering (Fig. 2b). To integrate the scATAC-seq data (Fig. 2c) with scRNA-seq clusters annotated by cell-type markers, we conducted Seurat Label Transfer (Fig. 2d). Briefly, scATAC-seq reads in promoters and gene bodies were counted to represent the open chromatin status of each gene as gene activities. From the gene expression level (scRNA-seq) and gene activity (scATAC-seq) data, the shared characters of the two data sets were extracted as anchors. Using these anchors, scRNA-seq clusters were transferred as a reference into scATAC-seq patterns. This integration ignored several regulatory factors, such as transcription factor binding and enhancers. We suggest that each layer of single-cell data sets is carefully analyzed in detail before different multiple layers are integrated. We may be successful in roughly integrating scRNA-seq and scATAC-seq at the cell type level (i.e., epithelial cells, immune cells) using Seurat and LIGER. However, integration focusing on detailed cellular states including unknown cell subpopulations and transition events that are only determined by the epigenome would be more difficult because these tools use scATAC-seq data as RNA-seq data, ignoring binary patterns of open chromatin data and complicated transcriptional regulation. Methods such as scAI^62^ have indicated the weakness of gene activity-based integration, and different approaches have been reported to overcome these weaknesses. The mouse lung data sets are provided in our DBKERO database (https://kero.hgc.jp/).

A different group developed single-nucleus droplet-based sequencing (snDrop-seq) for gene expression profiling and single-cell transposome hypersensitive site sequencing (scTHS-seq) for the analysis of chromatin accessibility in more than 60,000 human brain cells and integrated the two data sets^63^. They used a gradient boosting model (GBM) to associate differential accessibility with differential gene expression and to understand cell-type-specific transcriptional regulation in the human brain. This integration strategy is helpful for annotating cell types from both chromatin accessibility data and gene expression data and for understanding the association between transcriptional regulation and gene expression for each of the cell types. To identify the causes of mixed-phenotype acute leukemia, Granja et al.^64^ conducted CITE-seq (see below), scATAC-seq and scRNA-seq analysis. They integrated chromatin accessibility and gene expression data by using Seurat CCA and identified responsible transcription factors in leukemia.

### Multilayered sequencing from the same cells

**Fig. 3:**
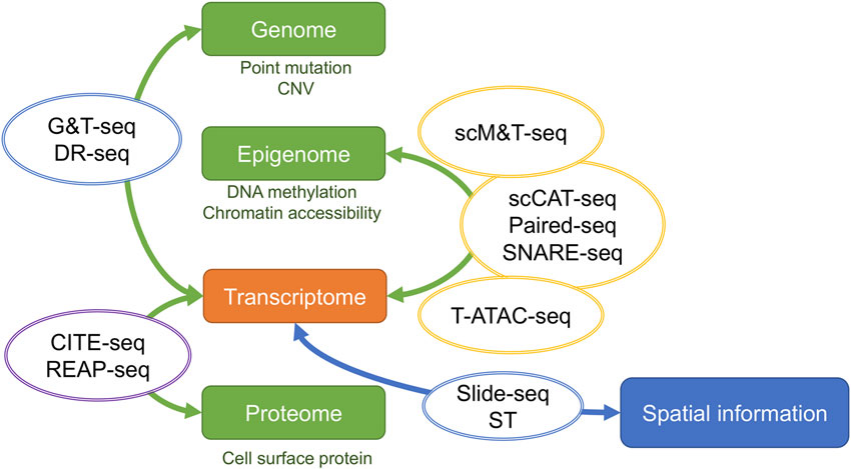
Multilayered single-cell sequencing. Representative single-cell multimodal sequencing methods. Genomic, epigenomic, and proteomic information can be simultaneously profiled with the transcriptome. Spatial information for a tissue section can also be obtained with gene expression data at the level of one to tens of cells. ST spatial transcriptomics (Visium).

Once an individual cell is used for the sequencing analysis of a single omics layer, we cannot profile different layers of omics information from the same cell. Methods that analyze two or more omics layers from a single cell have been reported^65^ (Fig. 3 and Table 4). G&T-seq^66^ and DR-seq^67^ were developed for simultaneously analyzing genomic DNA sequences and mRNA profiles. The copy number profile and expression profile accuracy of these methods is similar to that achieved via conventional WGA and WTA methods, respectively. scDam&T-seq^68^ measures both protein–DNA interactions and transcriptome profiles in the same cell and can thus couple transcriptional regulation analysis and gene expression analysis in individual cells by focusing on chromatin-associated proteins such as the lamina and Polycomb complex. As described above, the rapid development of scRNA-seq platforms has enabled us to easily obtain single-cell transcriptome profiles. However, it is still difficult to obtain single-cell genome sequences for joint analysis with transcriptome data from the same cell because no automatic platforms have been developed for the simultaneous measurement of the only two copies of genomic DNA and the 0.1–1 million mRNA molecules per cell, let alone for addressing the difficulty of avoiding dropout and detection bias. There are still only a small number of reports of the use of these methods.

**Table 4:** Multilayered sequencing from the same cells.

Method	Target	Cell isolation technique	Method feature	References
G&T-seq	Genome, transcriptome	FACS (96 well plate)	MDA/PicoPlex (WGA), SMART-seq2 (WTA)	^66^
DR-seq	Genome, transcriptome	Pipet (low throughput)	No physical separation of DNA and RNA	^67^
scM&T-seq	DNA methylation, transcriptome	Same as G&T-seq	Based on scBS-seq and G&T-seq	^80^
scDam&T-seq	Chromatin, transcriptome	FACS (384 well plate)	Based on DamID and CEL-seq	^68^
T-ATAC-seq	Open chromatin, TCR	C1 Single-Cell Auto Prep System	Based on scATAC-seq and TCR-seq	^69^
SNARE-seq	Open chromatin, transcriptome	Drop-seq (high throughput)	Tn5-DNA/mRNA captured by beads	^70^
scCAT-seq	Open chromatin, transcriptome	FACS (96 well plate)	Separation of nucleus and cytoplasm	^72^
CITE-seq	Surface protein, transcriptome	Drop-seq/Chromium (high throughput)	Protein detected by barcode-conjugated antibodies	^73^
REAP-seq	Surface protein, transcriptome	Chromium (high throughput)	Protein detected by barcode-conjugated antibodies	^74^

Transcript-indexed ATAC-seq (T-ATAC-seq)^69^ combines open chromatin profiling with the analysis of T cell receptor genes and thus analyzes epigenomic profiles in T cell clones. SNARE-seq^70^, Paired-seq^71^, and scCAT-seq^72^ enable the measurement of chromatin accessibility and whole-transcriptome profiles. In addition, 10× Genomics is scheduled to release the Single-Cell ATAC + Gene Expression platform for the simultaneous profiling of the epigenome and transcriptome by combining scRNA-seq with ATAC-seq (https://www.10xgenomics.com/product-updates/). We can expect that researchers will be able to access a simple protocol and platform for this purpose. Epigenomic landscapes determine the basic characteristics of cells, such as the cell lineage and differentiation state, while the transcriptome status represents the consequences of the cell conditions in a given state. Methods that can measure both transcriptome and open chromatin status in a single cell enable the elucidation of the direct link between transcriptome networks and their regulation, including the epigenome landscape and responsible transcription factors in each cell, resulting in an increasing number of reports and data sets arising from the simultaneous measurement of gene expression and ATAC-seq profiles.

For the simultaneous expression profiling of transcripts and cell surface proteins, CITE-seq^73^ and REAP-seq^74^ were developed, which are used mainly in immune cell analysis. Antibodies conjugated to barcode sequences are used to capture target cell surface proteins, and mRNAs and the barcode sequences of antibodies are analyzed for each cell. Feature Barcoding (10× Genomics) enables the combined profiling of targeted cell surface proteins with scRNA-seq via the Chromium system. The protocol is very simple and easily conducted: antibodies conjugated with each Feature Barcode oligo used to mark cell surface protein expression are mixed, single-cell separation, and amplification are conducted via the Chromium platform, and libraries of both cDNA and antibody-derived tags are constructed. Several cell types, especially immune cells, have historically been classified according to patterns of cell surface proteins. For example, naive, memory, and effector T cells are distinguished using CD45 isoform patterns (CD45RA/CD45RO antigens); however, these isoforms are not measured via general 3′ scRNA-seq, which indicates that information on the expression of cell surface markers may support the classification and interpretation of cell subsets. 10× Genomics also announced that they will release a method for the detection of intracellular proteins combined with gene expression profiling in a cell. The application of multilayered single-cell sequencing has expanded to include its combination with proteomics analysis.

## Conclusion

In this review, we summarized single-cell sequencing methods applied at the genome, epigenome and transcriptome levels and their combinations, even including proteome-level analysis. An increasing number of experimental and computational methods are being rapidly developed for single-cell analysis, and we need to understand the advantages and disadvantages of each of these methods. We can obtain various omics profiles from each individual cell and should utilize the obtained information to understand the heterogeneity of molecular profiles, their changes in a given population, and the interaction among cells, although the obtained data sets include high-dimensional and mostly sparse data and, thus, are not easy to handle. Multiomics data analysis from the same single cell is more reliable than the integration of single omics layers because less sampling bias and fewer batch effects are involved, as shown by CITE-seq, for example. However, it is still easier to obtain single-layered data from single cells, and their integration may allow more cost-effective and less time-consuming analysis to be achieved by utilizing publicly available data. The data coverage (sequencing depths and the number of detected genes/regions) may be better for single omics data because more sequencing reads are required to cover two or more layers in multiomics sequencing. We can utilize a combination of single and multilayered sequencing depending on the omics layers involved.

Furthermore, the results obtained with single-cell sequencing technologies lack spatial information because a tissue is dissociated into single cells before sequencing analysis. Recently, spatial transcriptome techniques in which gene expression analysis is conducted in tissue sections have been reported, where spatial information is retained via molecular barcoding; these include methods such as the Slide-seq^75^ and Visium (10× Genomics/Spatial Transcriptomics) approaches^76^. Using Visium, gene expression profiles from one to tens of cells can be measured in up to 5000 spots (55 μm diameter per spot) on a slide for each tissue section. A frozen tissue section with a 10–20 μm thickness is prepared on the slide with oligos containing spatial barcodes and UMIs. By sequencing the synthesized cDNA libraries, we obtained RNA-seq data for each local spot with spatial information. By comparing these data with an H&E-stained image, we can compare gene expression patterns with histopathological information. Although existing spatial transcriptome techniques are still not available at a single-cell resolution, they enable us to identify differential expression patterns depending on the condition of each local microenvironment within tissues. We need to not only deal with single-cell multiomics information but also integrate temporal and spatial information to understand the diverse omics features of each individual cell.
